# Predictors of radioiodine (RAI)-avidity restoration for NTRK fusion-positive RAI-resistant metastatic thyroid cancers

**DOI:** 10.1530/ETJ-23-0227

**Published:** 2024-05-09

**Authors:** Abdul Rehman Syed, Aakash Gorana, Erik Nohr, Xiaoli-Kat Yuan, Parthiv Amin MASc, Sana Ghaznavi, Debbie Lamb, John McIntyre, Markus Eszlinger, Ralf Paschke

**Affiliations:** 1 University of Calgary, Calgary, Alberta, Canada; 2 Arnie Charbonneau Cancer Institute, Cumming School of Medicine, University of Calgary, Alberta, Canada; 3 Alberta Precision Laboratories, Molecular Pathology Program, Cumming School of Medicine, University of Calgary, Calgary, Alberta, Canada; 4 Precision Oncology Hub Laboratory, Tom Baker Cancer Centre, Calgary, Alberta, Canada; 5 Department of Radiology, Cumming School of Medicine, University of Calgary, Calgary Alberta, Canada; 6 Arnie Charbonneau Cancer Institute, Department of Medicine, Section of Endocrinology, University of Calgary, Calgary, Alberta, Canada; 7 Department of Oncology, Cumming School of Medicine, and Arnie Charbonneau Cancer Institute, University of Calgary, Calgary, Alberta, Canada; 8 Departments of Medicine, Section of Endocrinology, Oncology, Pathology and Laboratory Medicine, Biochemistry and Molecular Biology and Arnie Charbonneau Cancer Institute, Cumming School of Medicine, University of Calgary, Calgary, Alberta, Canada

**Keywords:** radioiodine uptake reinduction, radioiodine avidity restoration, NTRK fusion gene thyroid cancer, radioiodine resistance, larotrectinib

## Abstract

**Context:**

Two-thirds of metastatic differentiated thyroid cancer (DTC) patients have radioiodine (RAI)-resistant disease, resulting in poor prognosis and high mortality. For rare *NTRK* and *RET* fusion-positive metastatic, RAI-resistant thyroid cancers, variable success of re-induction of RAI avidity during treatment with *NTRK* or *RET* inhibitors has been reported.

**Case presentation and results:**

We report two cases with RAI-resistant lung metastases treated with larotrectinib: an 83-year-old male presenting with an *ETV6::NTRK3* fusion-positive tumor with the *TERT* promoter mutation c.-124C>T, and a 31-year-old female presenting with a *TPR::NTRK1* fusion-positive tumor (and negative for *TERT* promoter mutation). Post larotrectinib treatment, diagnostic I-123 whole body scan revealed unsuccessful RAI-uptake re-induction in the *TERT*-positive tumor, with a thyroid differentiation score (TDS) of −0.287. In contrast, the *TERT*-negative tumor exhibited successful I-131 reuptake with a TDS of −0.060.

**Conclusion:**

As observed for RAI-resistance associated with concurrent *TERT* and BRAF mutations, the co-occurrence of *TERT* mutations and *NTRK* fusions may also contribute to re-sensitization failure.

## Graphical abstract


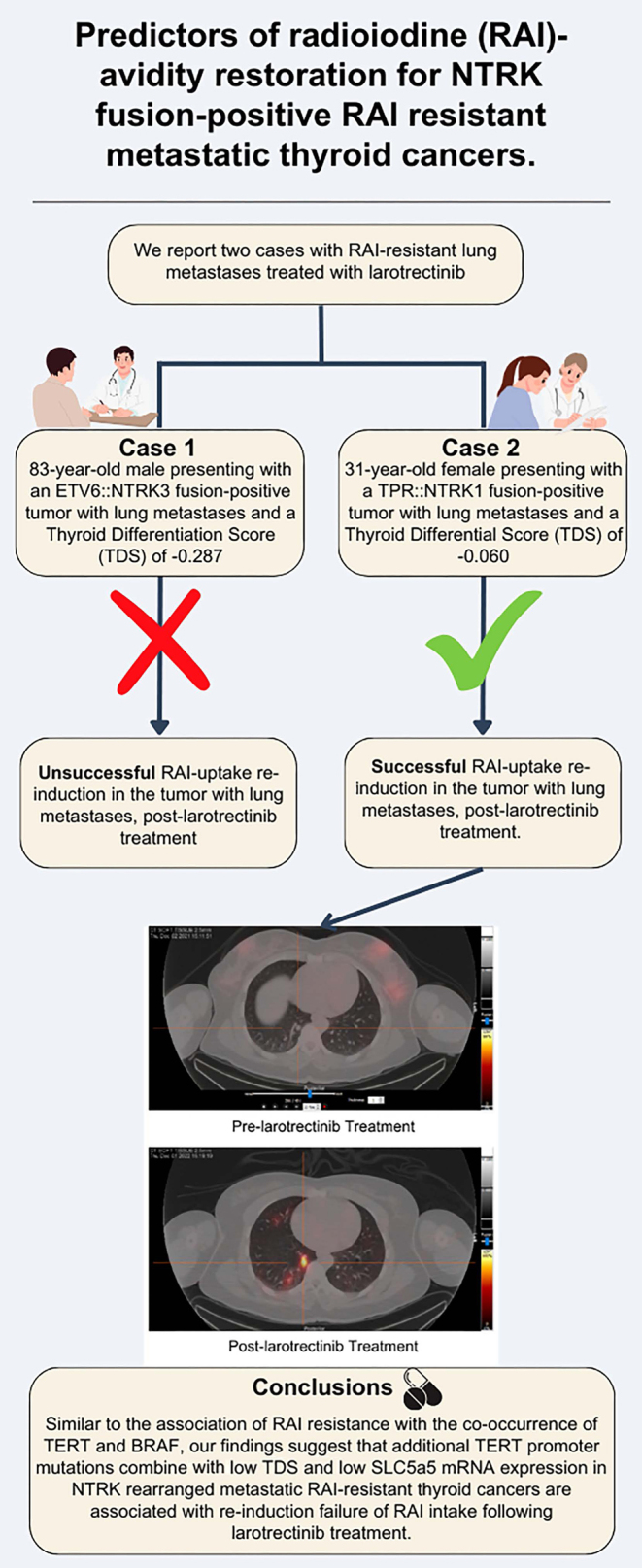


## Introduction

Papillary thyroid carcinoma (PTC) typically has an excellent clinical prognosis. However, 10% of cases of differentiated thyroid cancers (DTC) will progress to a metastatic stage after receiving initial treatment (1). Two-thirds of metastatic DTCs lose their ability to uptake radioiodine (RAI) due to oncogene driver mutations silencing thyroid iodide-metabolizing genes, causing them to be RAI-resistant or refractory (1, 2, 3). Patients with RAI-refractory metastatic DTC have a poor prognosis, with a mortality of approximately 90% within 10 years of diagnosis (4).

Data from The Cancer Genome Atlas (TCGA) investigating nearly 500 PTCs confirmed activation of the mitogen-activated protein kinase (MAPK) pathway for the large majority of PTCs (5). Among this cohort, *BRAF V600E* mutation was present in nearly 60% of PTCs, followed by *HRAS* and *NRAS* mutations in 10% of cases and *RET* fusions in ~5% of cases (5). Therapeutic approaches that inhibit specific mediators, first demonstrated with selumetinib, a MAPK pathway inhibitor, and subsequently with BRAF mutation inhibitors, have demonstrated the ability to restore RAI uptake for patients with metastatic RAI-refractory thyroid cancer (6, 7). Recently, this strategy was extended to rarer oncogenic fusion genes like *RET* and *NTRK1/2/3* rearrangements using the RET inhibitor selpercatinib and the NTRK inhibitor larotrectinib (6, 8, 9). The first instance of RAI uptake restoration leading to a structural response to RAI treatment was reported in a 64-year-old female adult with an *EML4:NTRK3* rearrangement-positive PTC with synchronous lymph and lung metastases (8). Restoration of RAI uptake was also observed in a metastatic *TPR::NTRK1* fusion-positive pediatric PTC; however, the patient did not undergo RAI therapy due to the protocol followed in the context of participation in a clinical trial (10). Recently, larotrectinib was reported to re-induce RAI uptake and subsequent RAI treatment led to a partial response according to RECIST at 3 months post RAI treatment in two of three female adults with metastatic, acquired and primary RAI-resistant *NTRK*-rearranged PTC (11). The reasons for the unsuccessful RAI re-sensitization in the third patient are unknown. In *BRAF* or *RAS* mutated or wildtype metastatic, RAI-resistant thyroid cancers treated with BRAF inhibitors and MEK inhibitors, re-induction of RAI uptake can only be achieved in 50–71% according to a recent systematic review of seven studies (12) and 60–95% in another recent study (13, 14). In a few patients, mutations in SWI/SNF genes were identified as the reason for the failure of RAI re-induction upon MAPK inhibition (15).

There is a compelling need to identify patients most likely to fail RAI re-sensitization treatments, as opposed to the current practice of empirically treating *BRAF*, *RAS* mutated, or *RET* or *NTRK*-rearranged metastatic RAI-resistant patients with RAI after MAPK inhibition irrespective of the tumor genotype. *TERT* promoter mutations were shown to be strongly associated with loss of RAI avidity and impairment of the iodide-metabolizing machinery in recurrent PTC (16). Therefore, we have analyzed the implication of *TERT* promoter mutations and the expression of iodide metabolizing genes in two patients with successful and unsuccessful RAI re-sensitization after treatment with the NTRK inhibitor larotrectinib.

## Patients

### Case 1

Case 1 is an 83-year-old male. In June 2017, the patient had a chest X-ray following an upper respiratory tract infection, which incidentally revealed multiple pulmonary nodules. Subsequent [18F]Fluorodeoxyglucose (^18^F-FDG) PET-CT revealed ~40 bilateral lung metastases, ranging from 5 mm to 14 mm. Synchronous thyroid nodules were discovered, and a 4.1 × 2.8 cm thyroid nodule biopsy was positive for follicular variant PTC. He is in otherwise good health. He has not been exposed to radiation or chemotherapy, nor does his family have any documented cases of hereditary cancer syndrome. In November 2017, a total thyroidectomy was performed with minimal extrathyroidal extension toward the trachea and positive for vascular invasion but no lymphatic or perineural invasion. Cancer staging of the mixed follicular, classical variant PTC was determined to be T4bNxM1 in accordance with the American Joint Committee on Cancer (AJCC), 8th edition (17), reflecting lung metastases and categorizing the patient as having a high risk of recurrence.

**Figure 1:**
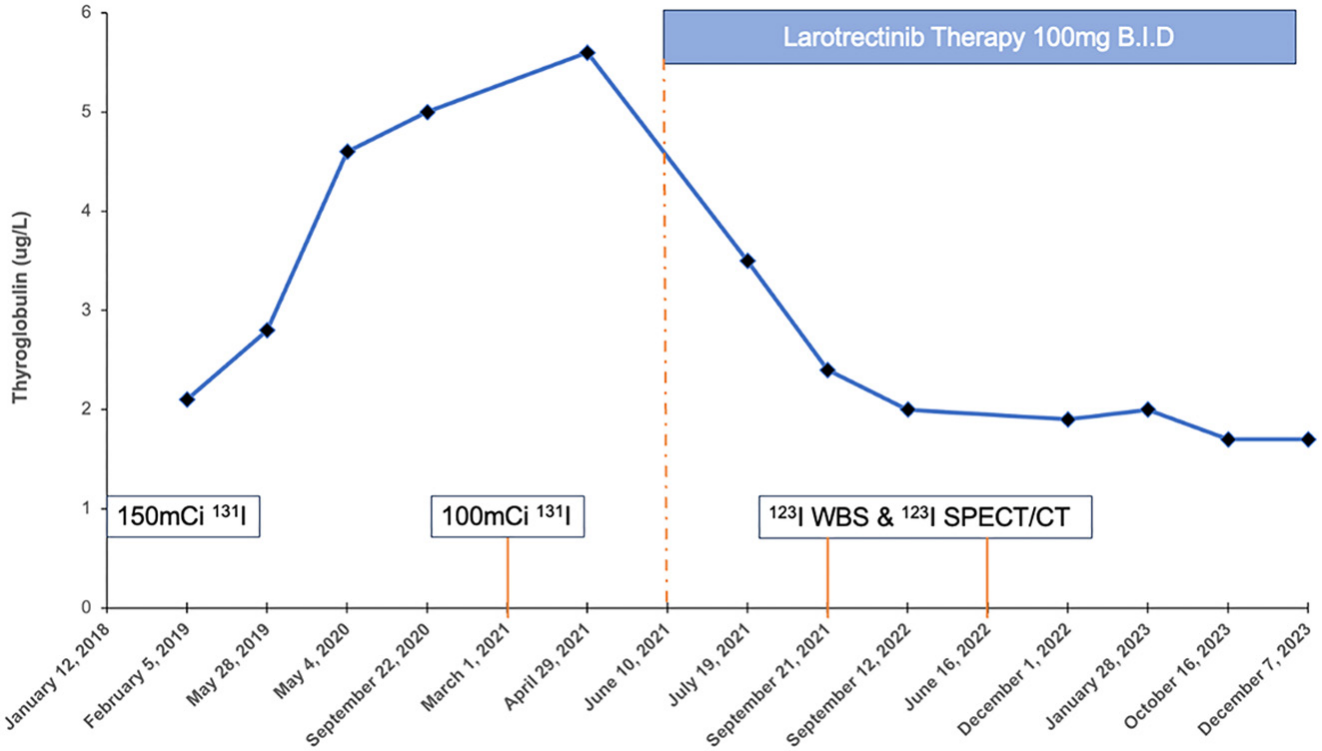
Tg values with suppressed TSH for case 1. RAI treatments are indicated. Case 1 received his first 150 mCi (5550 MBq) of ^131^I in January 2018, followed by another 100 mCi of ^131^I in March 2021. Two diagnostic ^123^I WBS and SPECT/CTs were performed in September 2021 and June 2023, respectively. Larotrectinib treatment began in June 2021.

In January 2018, he received 150 mCi (5550 MBq) of ^131^I for thyroid ablation with subsequent post-^131^I therapy whole-body scan(WBS) indicating RAI accumulation in the thyroid bed and a low-grade RAI accumulation within the lungs. In August 2018, a chest CT scan exhibited a general decrease in the size of the lung metastases, concomitant with a decrease in Tg levels from 3.3 µg/L in February 2018 to 1.4 µg/L in August 2018. However, between August 2018 and September 2020, Tg levels began to uptrend from a nadir of 1.4 µg/L to 5.0 µg/L (Fig. 1). Additionally, there was ~0.25 cm growth within the indexed pulmonary metastases detected on chest CT, while a neck ultrasound was negative for recurrence.

Further molecular analysis with the Oncomine Comprehensive Assay v3 (OCAv3) and ThyroSPEC (18, 19) revealed an *ETV6::NTRK3* fusion-positive tumor harboring an additional *TERT* promoter mutation c.-124C>T, with no other genetic alterations. The patient began larotrectinib 100 mg twice daily in June 2021.

### Case 2

A previously healthy 31-year-old female was diagnosed with PTC detected at 20 weeks’ gestation. She observed a neck lump 3 years prior, with no size alterations since its discovery. During her pregnancy, the patient had bilateral FNA biopsies of the thyroid performed due to a right lower lobe nodule measuring 3.7 × 2.3 cm, which showed microcalcifications and hypervascularity, as well as a 1.3 × 0.9 cm lymph node on the left side of her neck confirming classical PTC.

In March 2016, 1 month *post partum*, she underwent a total thyroidectomy, central and bilateral cervical lymph node dissection, and partial tracheal resection. Pathology evaluation confirmed classical variant PTC, with the largest tumor on the right lobe being 3.3 cm. Post-surgery pathology evaluation revealed 9 of 18 resected lymph nodes examined were positive for metastatic PTC, the largest metastasis being 2.0 cm. Moreover, pathology also confirmed microscopic extrathyroidal extension into skeletal muscles and vascular invasion**.**She received 100 mCi (3700 MBq) of ^131^I in June 2016.

Initially, in 2016, case 2 demonstrated a negative ^131^I WBS post RAI therapy as the scan displayed focal RAI uptake in the thyroid bed but no RAI-avid distant metastases. However, an increase in Tg prompted a comprehensive CT scan of the whole body, including a targeted scan of the head and neck with ^18^F-FDGPET in March 2018 to investigate further disease progression. The scans did not reveal any significant ^18^F-FDG uptake indicative of active disease, but they did identify numerous bilateral pulmonary nodules suspicious of metastatic spread, characterized by only low-grade ^18^F-FDG uptake. These nodules were scattered, primarily located in the lower lobes, among which two target lesions were identified. The first target lesion (target lesion 1) was situated at the medial basal region of the right lower lobe, measuring 1.2 × 1.1 cm, while the second lesion (target lesion 2) was the largest in the left lower lobe located at the anteromedial basal region measuring 0.8 × 0.8 cm. A core biopsy of the right lung nodules in June 2018 confirmed metastatic PTC. This led to a re-staging of the PTC to T4aN1bM1, in accordance with the AJCC, 8th edition (17), reflecting lung metastases.

Further, ThyroSPEC study of the primary tumor demonstrated a *TPR::NTRK1* fusion-positive tumor (without *TERT* promoter mutations). With evidence of a *TPR::NTRK1* fusion-positive tumor, the patient began treatment with larotrectinib at a dose of 100 mg twice daily in June 2022.

## Methods

### Larotrectinib treatment

Health Canada has authorized larotrectinib for the treatment of adults and children with solid tumors that exhibit NTRK gene fusion, provided that these tumors do not have a known acquired resistance mutation. It is applicable for patients whose tumors are either metastatic or in cases where surgical removal would likely cause significant harm, and who lack other effective treatment options.

In case 1, the patient began larotrectinib 100 mg twice daily starting in June 2021. With Synthroid 175 mg, TSH suppression was achieved.

In case 2, the patient began larotrectinib 100 mg twice daily starting in June 2022. With Synthroid 125 mg, TSH suppression was achieved.

**Figure 2:**
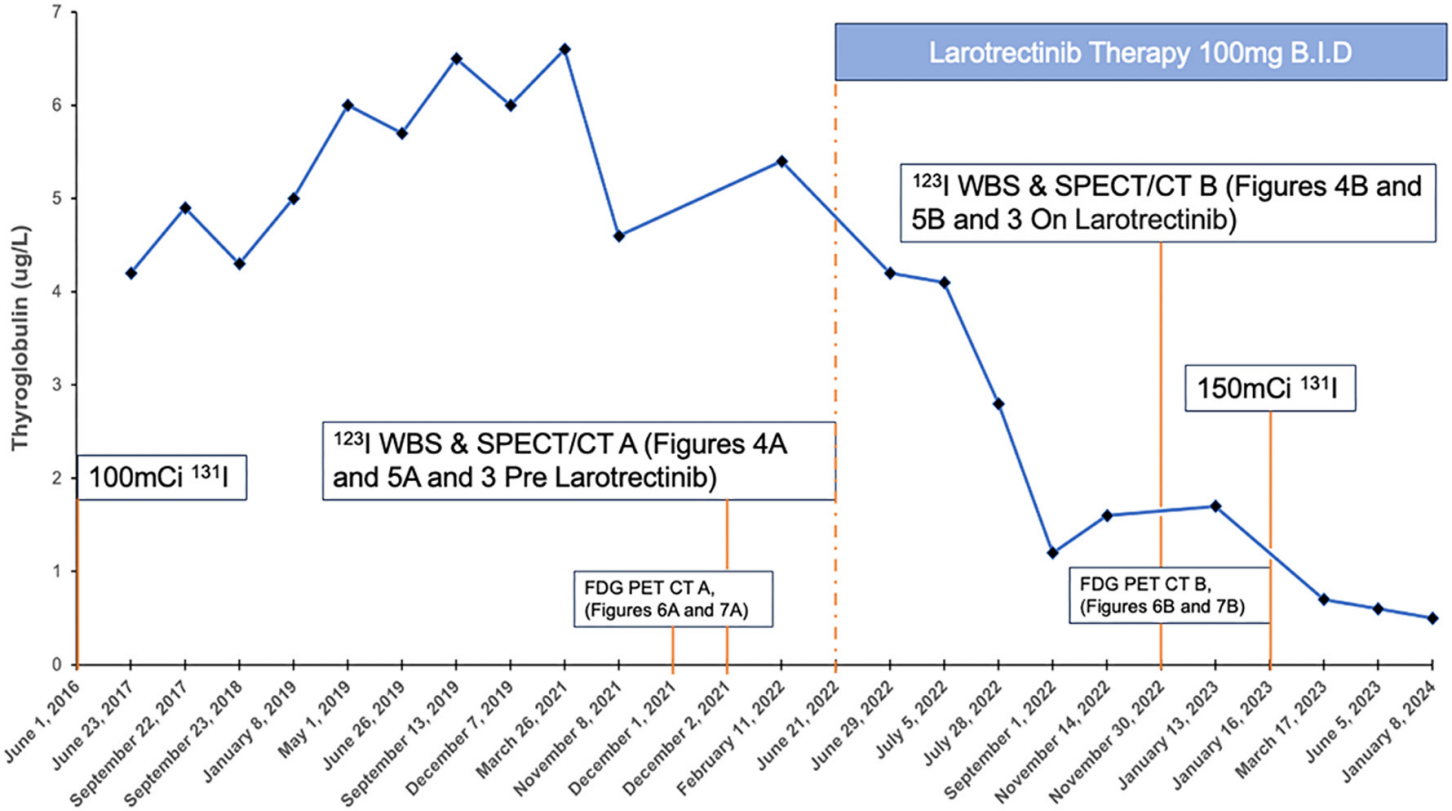
Tg values obtained with suppressed TSH recorded over time for case 2. ^123^I RAI treatments, ^123^I WBS and SPECT/CTs, and FDG PET-CTs are indicated. Case 2 received her first 100 mCi (3700 MBq) of ^131^I in June 2016, followed by a diagnostic ^123^I WBS (Fig. 3, pre-larotrectinib) and SPECT/CT A (Figs. 4A and 5A) in December 2021. Larotrectinib began in June 2022, followed by a 6-month on treatment diagnostic ^123^I WBS (Fig. 3, on larotrectinib) with SPECT/CT B (Figs. 4B and 5B) obtained on the same day. The evidence for reinduction of radioiodine uptake supported the repeat ^131^I RAI therapy in January 2023.

For both cases, at no point was larotrectinib paused during RAI treatment. Tg was monitored with serial follow-up appointments (Figs. 1 and 2).

#### ThyroSPEC

ThyroSPEC formalin-fixed, paraffin embedded (FFPE) analysis was performed as previously described by Eszlinger *et al*. (18) As part of the analysis, DNA and RNA are isolated from FFPE tissue of the primary tumor resected during the total thyroidectomy, pre-larotrectinib treatment.

#### RNAseq and determination of thyroid differentiation score

For RNAseq, 100 ng total RNA extracted from macrodissected FFPE material of the primary tumors were used to construct RNASeq libraries following the Illumina RNA Prep with Enrichment Tagmentation protocol. RNA was denatured, and first-strand cDNA was synthesized, followed by second-strand synthesis. cDNA was then fragmented using bead-linked transposomes, and adapter sequences were added. Libraries were then cleaned and normalized before hybridizing to exome probes. Hybridized probe libraries were captured, washed, and amplified to enrich the target library. The enriched library was cleaned, and quality metrics were assessed before sequencing on a NextSeq 500 (Illumina). Thyroid differentiation score (TDS) was derived from primary tumor RNAseq. The mean log2 fold changes for mRNASeq read counts for 16 thyroid differentiation genes were determined as described in the TCGA study (5).

## Results

### Larotrectinib treatment

Case 1: One-year post larotrectinib start, Tg levels decreased from a pre-treatment value of 5.6 µg/L recorded in April 2021 to 1.7 µg/L obtained in October 2023 (Fig. 1), while a ^18^F-FDG PET-CT scan demonstrated reduced metabolic activity and size reduction of pulmonary metastases. However, diagnostic ^123^I WBS at 3 and 12 months post larotrectinib treatment were negative for re-induction of RAI uptake; thus, the patient did not receive further RAI treatment.

Case 2: Larotrectinib at a dose of 100 mg twice daily started in June 2022 yielded favorable radiological and biochemical responses over the next year. The patient’s Tg levels began to steadily decline from a pre-treatment value of 6.0 µg/L in May 2022 to 1.9 µg/L in January 2023 (Fig. 2).

**Figure 3:**
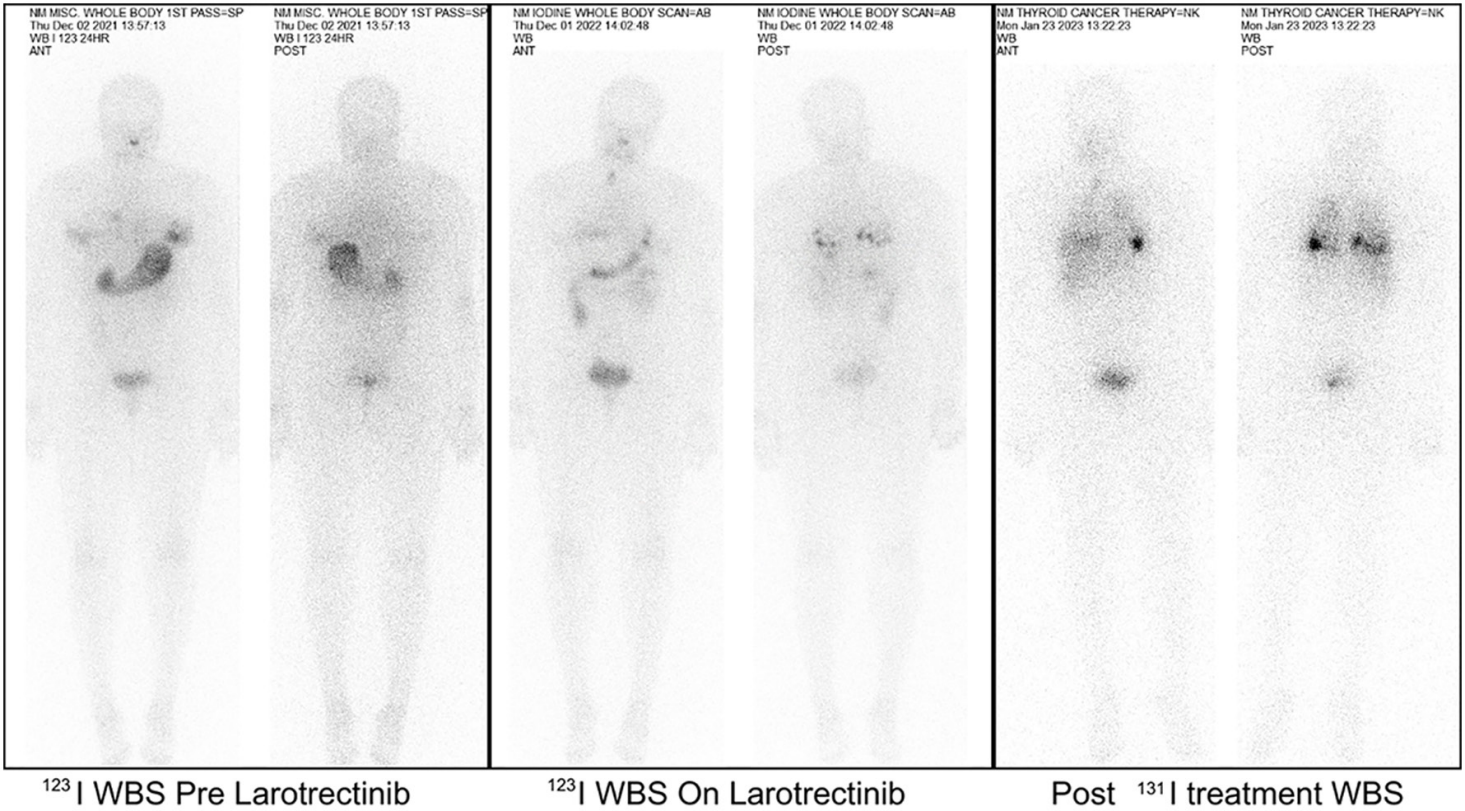
Chronological comparison of whole-body scans (WBS) in anterior and posterior viewing panels for *TPR::NTRK1* fusion-positive and *TERT*-negative case 2. Pre-diagnostic ^123^I WBS displays the diagnostic ^123^I WBS obtained pre-larotrectinib treatment in December 2021. Post-diagnostic ^123^I WBS exhibits the diagnostic ^123^I WBS obtained at 6 months since the initiation of larotrectinib treatment in December 2022, which showed reinduction of radioiodine uptake in comparison to the December 2021 scan. Post-^131^I treatment WBS on the right is the post-150 mCi (5550 MBq) ^131^I treatment WBS at 7 months post larotrectinib treatment with re-induction of RAI in pulmonary metastases.

In December 2022, a diagnostic ^123^I WBS revealed RAI uptake in the lung metastases (^123^I WBS on larotrectinib, Fig. 3); this was a significant development compared to the ^123^I WBS conducted pre-larotrectinib treatment in December 2021, which showed no RAI uptake in the thyroid bed or in the pulmonary metastases (^123^I WBS pre-larotrectinib, Fig. 3). This finding supported the decision to administer a further round of RAI therapy, leading to the patient receiving 150 mCi (5550 MBq) ^131^I therapy in January 2023 (post-^131^I treatment WBS, Fig. 3).

**Figure 4:**
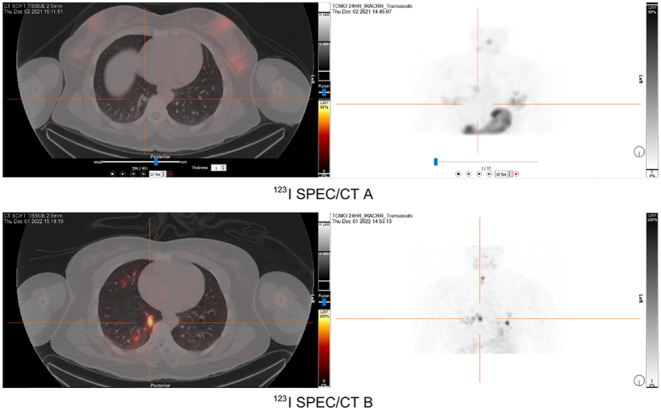
Case 2 ^123^I SPECT/CTs (left panels) and maximum intensity projection (MIP) (right panels) obtained before and after larotrectinib treatment for target lesion 1. ^123^I SPECT/CT A represents pre-larotrectinib ^123^I SPECT/CT and MIP of the chest in December 2021. In comparison, ^123^I SPECT/CT B was obtained post larotrectinib therapy, indicating increased ^123^I uptake, in December 2022.

**Figure 5:**
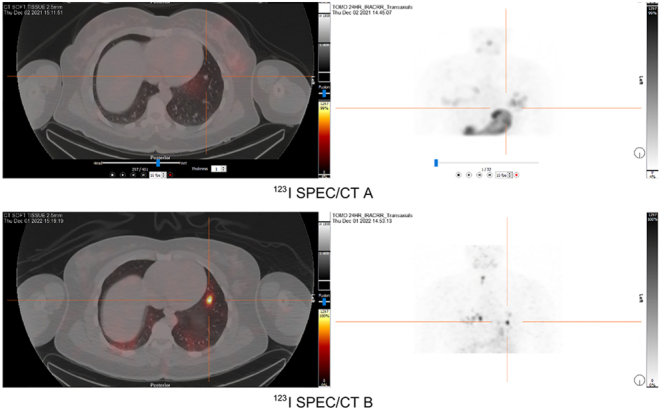
Case 2 ^123^I SPECT/CTs (left panels) and maximum intensity projection (MIP) (right panels) obtained before and after larotrectinib treatment for target lesion 2. Pre-larotrectinib therapy ^123^I SPECT/CT and MIP conducted in December 2021 are represented in ^123^I SPECT/CT A. In comparison, ^123^I SPECT/CT B was obtained post larotrectinib therapy, indicating increased ^123^I uptake, in December 2022.

Diagnostic imaging with ^123^I SPECT/CT scans was conducted both before and during larotrectinib treatment. The initial ^123^I SPECT/CT scan in December 2021 (Figs. 4A and 5A) showed no RAI uptake before treatment. However, a follow-up ^123^I SPECT/CT B (Figs. 4B and 5B) conducted 6 months into larotrectinib therapy, in December 2022, revealed a significant reduction in the size of target lesions along with increased ^123^I uptake, indicating a positive response to the treatment.

**Figure 6:**
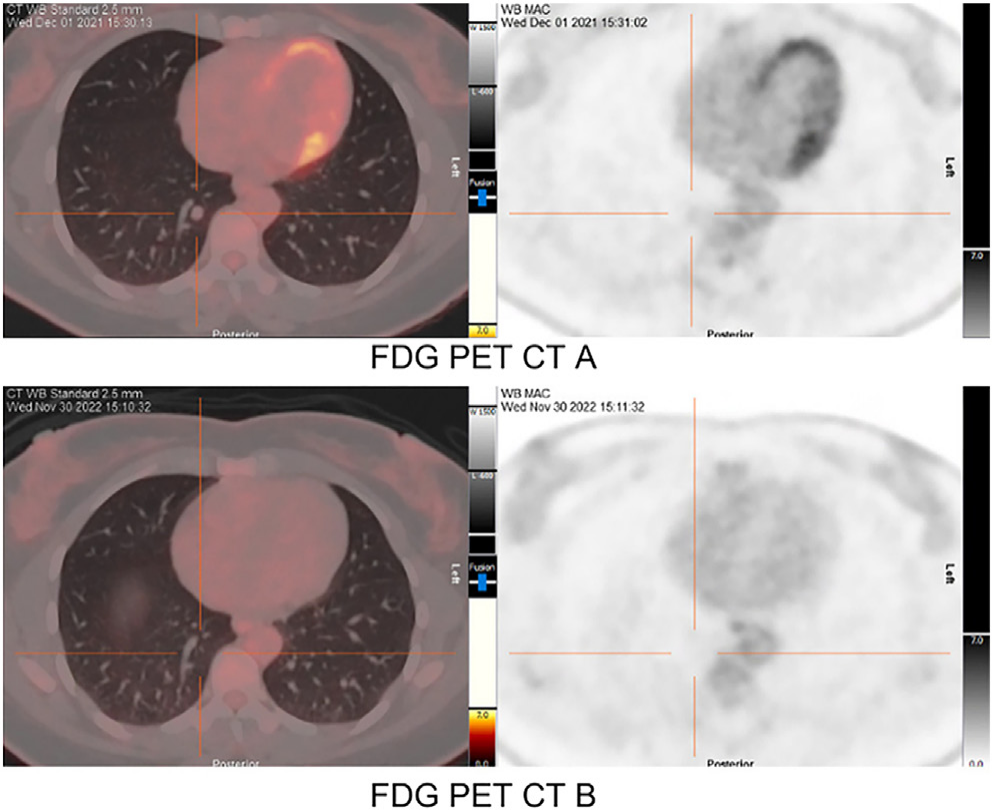
Case 2 FDG PET-CTs (left panels) and maximum intensity projection (MIP) (right panels) obtained before and after larotrectinib treatment for target lesion 1. Pre-larotrectinib therapy FDG PET conducted in December 2021 is represented by FDG PET A. In comparison, FDG PET B was obtained post larotrectinib therapy in November 2022, demonstrating a reduction in size and decreased FDG-PET avidity of the target lesion.

**Figure 7:**
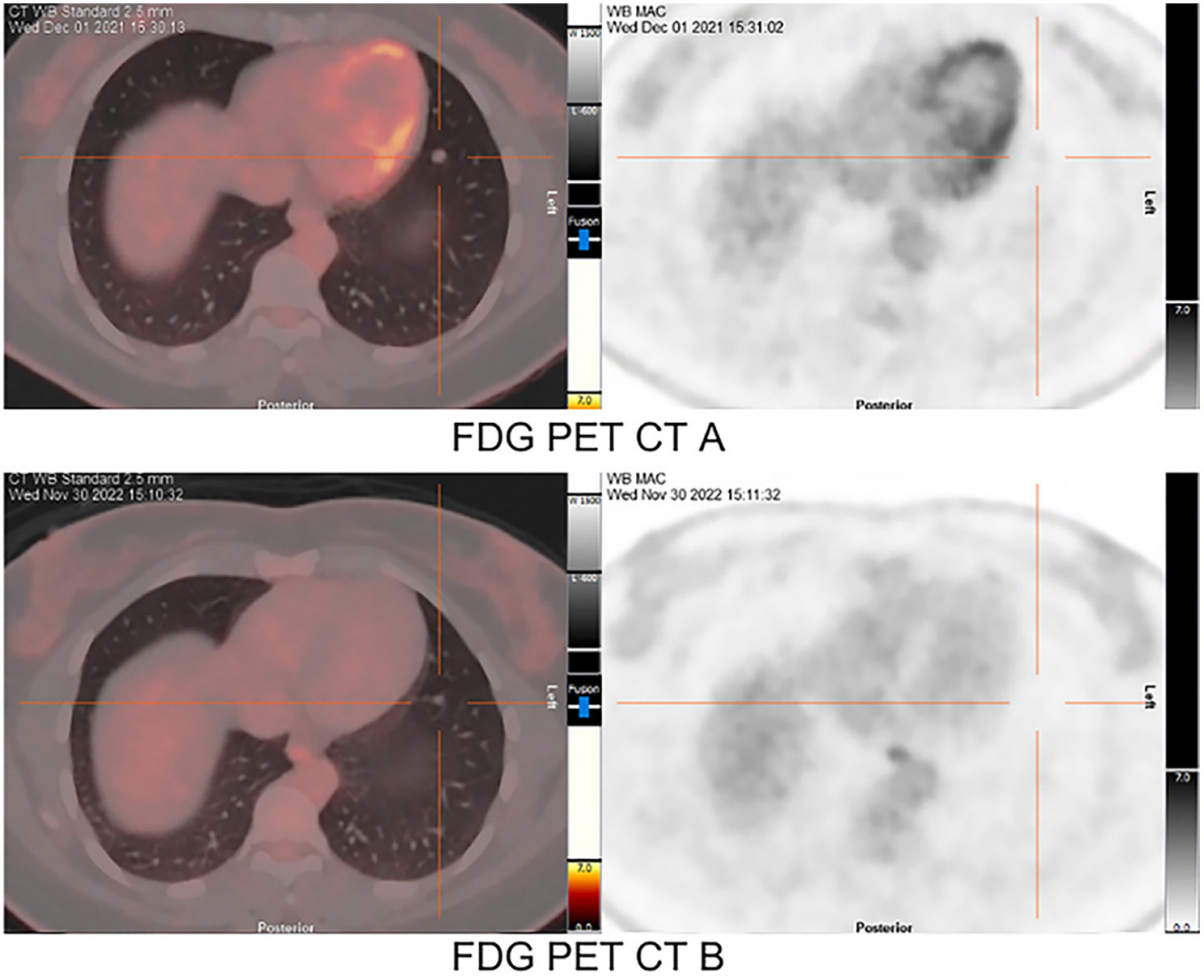
Case 2 FDG-PETs and maximum intensity projection (MIP) (right panels) obtained before and after larotrectinib treatment for target lesion 2. Pre-larotrectinib therapy FDG PET CT and MIP of the chest conducted on December 2021 in FDG-PET A. In comparison, FDG-PET B was obtained post larotrectinib treatment, indicating a reduction in size and decreased FDG-PET avidity of the target lesion, in November 2022.

To provide additional support for the decision to proceed with another round of ^131^I therapy, ^1^ ^8^F-FDG PET/CT imaging was also conducted. The pre-treatment scan in December 2021 showed low-grade metabolic activity within known pulmonary metastases (FDG PET-CT A, Figs. 6 and 7). Contrastingly, the ^1^ ^8^F-FDG PET/CT scan performed after 6 months of larotrectinib treatment demonstrated a favorable anatomical and metabolic response (FDG PET-CT B, Figs. 6 and 7). This improvement was characterized by a decrease in the size of targeted lesions and the elimination of the previously observed low-grade metabolic activity, in addition to a reduced FDG-PET avidity in the target lesions.

Finally, post-^131^I WBS displayed low-grade metabolic activity in the right thyroid bed and multiple RAI-avid lung metastases with significant uptake, confirming restoration of RAI-avidity (post-^131^I treatment WBS, Fig. 3).

In addition, a chest CT conducted in May 2022 (1 month before larotrectinib treatment) visualized target lesion 1 measured at 8 × 6 mm, while the target lesion 2 measured at 8 × 7 mm. By June 2023, a follow-up chest CT scan demonstrated that these target lesions had significantly reduced in size, with the first target lesion decreasing from 8 × 6 mm to 5 × 4 mm and the second target lesion from 8 × 7 mm to 5 × 5 mm, respectively.

At the time of this report, both patients continue to tolerate larotrectinib without toxicity.

#### RNAseq and determination of thyroid differentiation score

**Figure 8:**
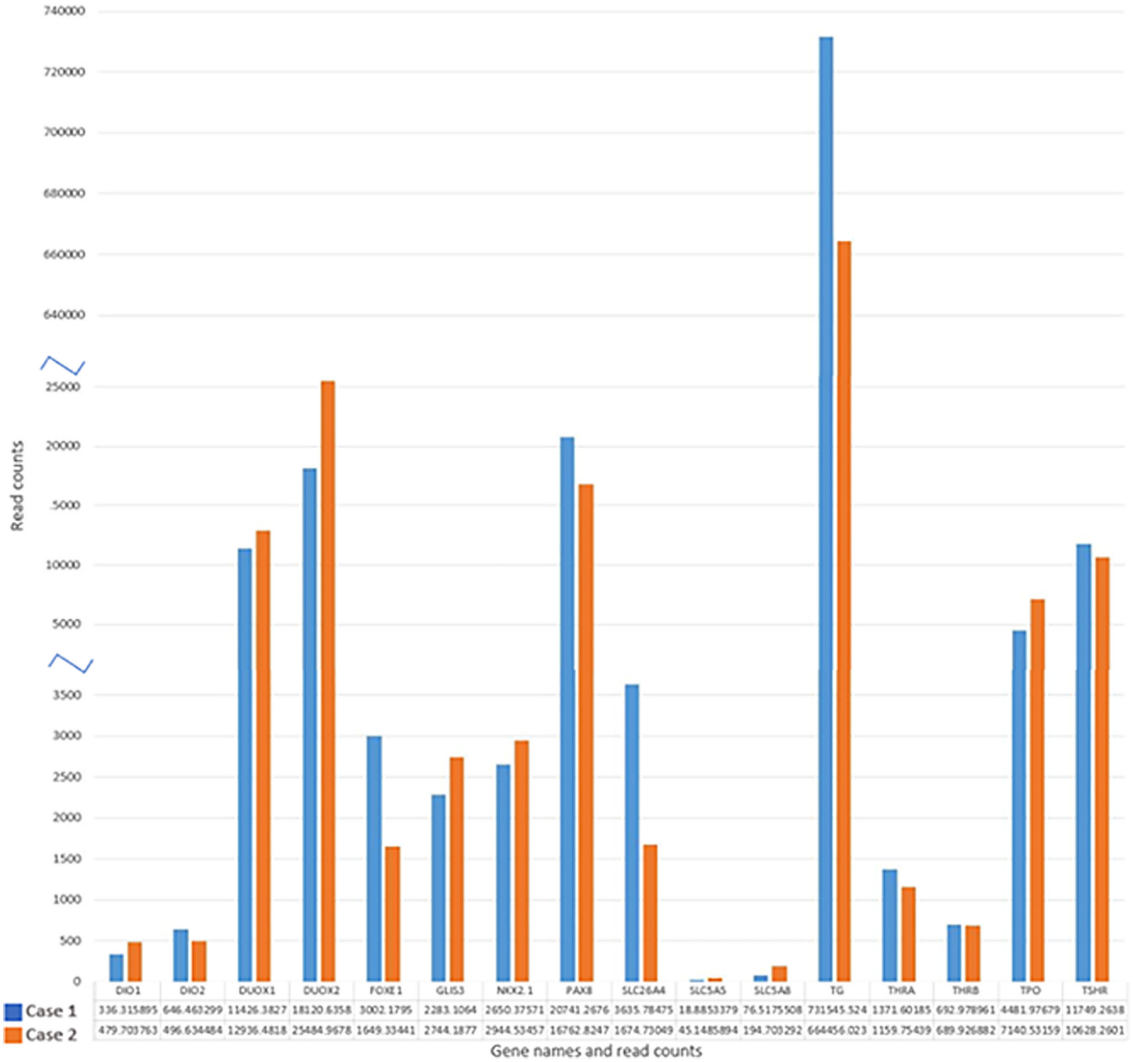
Normalized mRNA expression values for TDS genes for case 1 and case 2. The values in the figure indicate the number of counts for the respective genes.

**Figure 9:**
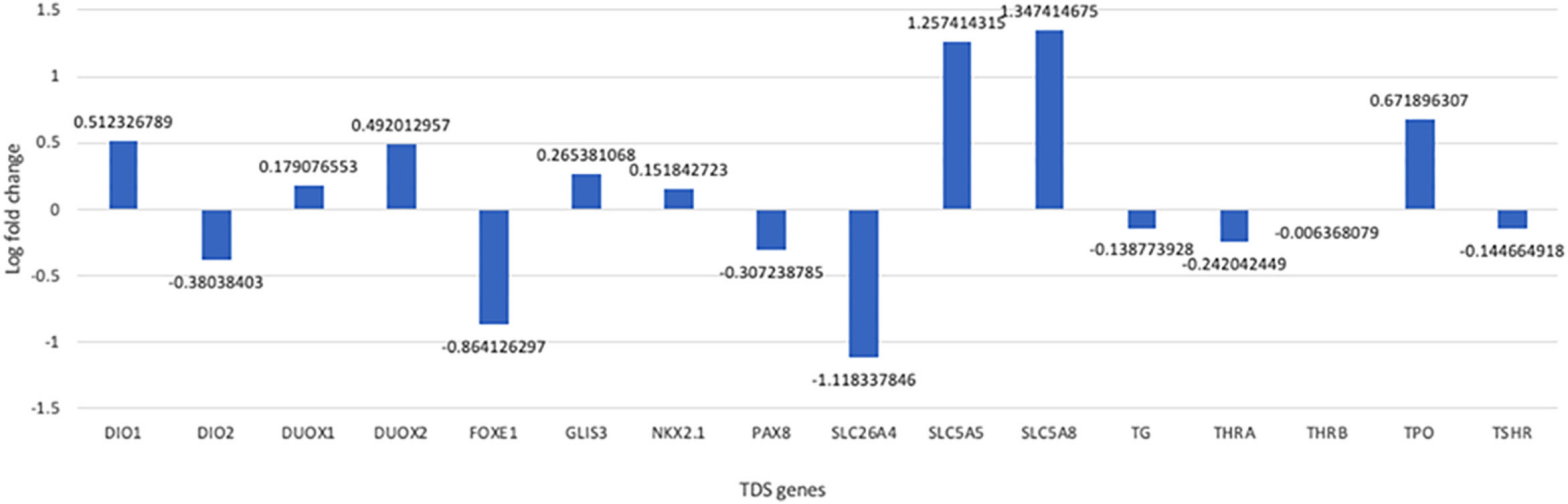
Log 2 fold changes (log 2 expression values of case 2 divided by expression values of case 1) for mRNA expression of TDS genes.

The mean log2 of fold changes for mRNASeq read counts of 16 thyroid differentiation genes, as described in the TCGA study (5), was −0.287 for the *TERT* positive and −0.060 for the *TERT* negative tumor, respectively. The apical iodide transporter (*SLC5A8*) and the sodium–iodide symporter (*SLC5A5*) gene expression was characterized by a 2.5-fold and a 2.4-fold upregulation of normalized expression values in the *TERT*-negative compared to *TERT*-positive tumor, respectively, whereas Pendrin (SLC5A8) showed increased expression (Fig. 8). Log2 fold changes are given in Fig. 9.

## Discussion

In summary, mutation and RNA expression analysis of the primary *NTRK*-rearranged tumors detected a *TERT* promoter mutation and a low TDS, with downregulation of the apical iodide transporter (*SLC5A8*) and the sodium–iodide symporter (*SLC545*) gene expression in the primary tumor of a patient without re-induction of RAI uptake (Fig. 8). In contrast, in the patient showing re-induction of RAI uptake following treatment with larotrectinib, her tumor was negative for *TERT* promoter mutations and showed high TDS with no downregulation of the apical iodide transporter (*SLC5A8*) and the sodium–iodide symporter (*SLC5A5*) gene expression (Fig. 8). Our findings suggest a possible association between additional TERT promoter mutations, alongside low TDS and *SLC5A5* mRNA expression in NTRK-rearranged metastatic RAI-resistant thyroid cancers and the re-induction failure of RAI uptake following larotrectinib treatment. However, given that these observations stem from a study of only two patients, further research is essential to validate these findings.

The potential mechanism of action for the re-differentiating effect of larotrectinib might be similar to that of MAPK inhibitors (10). *In vitro* experiments demonstrated larotrectinib-induced restoration of RAI uptake was mediated by sodium/iodide symporter re-expression. This re-differentiating effect was also described for *RET* rearrangements (9, 10). Corroborating our TDS analysis, *in vitro* studies conducted by Lee *et al*. (10) also found that the expression of several genes, notably *SLC5A8* and especially *SLC5A5 –* which are critical for RAI uptake in cells – were expressed at very low levels in patients with two RAI-refractory PTC. This underexpression of *SLC5A5* could potentially account for RAI resistance observed in these tumors. Our study findings also revealed a noteworthy 2.5-fold upregulation of *SLC5A8* and a 2.4-fold upregulation of *SLC5A5* in the *TERT*-negative tumor as compared to the *TERT*-positive tumor (Fig. 9).

The lower mRNA expression of the *SLC5A5* gene pre-larotrectinib therapy in case 1 and the negative post-RAI treatment WBS may be attributed to the presence of the *TERT* promoter mutation. An analysis of *SLC5A5* expression on 378 primary PTCs by Tavares *et al*. (20) demonstrated a significantly lower *SLC5A5* expression in PTCs that harbored *TERT, BRAF,* or especially dual *BRAF and TERT* mutations compared to wild-type PTCs (20). In another retrospective study, there was a loss of RAI avidity in 97% of all patients with recurrent disease and the presence of co-existing *BRAF* and *TERT* mutation, pointing to a potential synergistic effect (16). The authors also analyzed PTC data in TCGA database for the expression of the thyroid iodide-metabolizing genes, including *SLC5A5*, *TSHR*, *TPO*, *TG*, and *PAX8* in 386 PTC samples that had information available for the analysis and showed lower expression in the *TERT*-positive group than the *TERT*-negative group (16).

The TCGA study identified *TERT* mutations in 9.4% of 384 tumors (5). The impact of *TERT* mutation was further investigated in patients with distant DTC metastases (21). Of the 66 patients with distant DTC metastases, 15 harbored a *TERT* mutation, and a rising Tg was observed in 14 patients. Notably, all these patients were classified as RAI-refractory and were associated with an older mean age (~58) at diagnosis, larger tumors, and a greater likelihood of *BRAF V600E* mutation.

Although the 83-year-old male did not display reinduction of RAI uptake after treatment with larotrectinib, there was a clear clinical and radiological benefit of larotrectinib. This observation is consistent with the respective description of the patient with a lack of re-sensitization post larotrectinib treatment by Groussin *et al*. (11) and re-demonstrates a difference between the antitumoral and the re-differentiating effect of the NTRK inhibitor.

The Ion AmpliSeq Cancer Hotspot Panel v2 (Life Technologies) was used for the analysis of the previously described *NTRK*-rearrangement positive metastatic RAI-resistant PTC with failure of RAI re-induction post larotrectinib therapy (11). *TERT* is not covered by this panel (personal communication with Dr Oliver Huillard) and was instead covered on ThyroSPEC testing. Unfortunately, five single-arm (6, 7, 14, 22, 23) and three uncontrolled retrospective studies (24, 25, 26) on MAPK inhibition of metastatic RAI-resistant thyroid cancer with *BRAF*, *RAS,* or no detected driver mutation for RAI re-sensitization do not provide any information regarding *TERT* mutation analysis. Therefore, in addition to the analysis of the driver mutation, future RAI re-sensitization studies should provide information regarding the *TERT* mutation status of the primary tumors, and if possible, the *TDS* gene expression.

## Declaration of interest

ARS, AG, EN, X-KY, PA, SG, DL, and JM have no disclosures to make. ME and RP receive licensing fees for ThyroSPEC.

## Funding

RP received financial support for Research and Advisory Board honoraria from Bayerhttp://dx.doi.org/10.13039/100004326.

## Statement of ethics

Written informed consent for publication of their clinical details and clinical images was obtained from the patient.

## Author contribution statement

AR: visualization and writing (original draft). AG: analysis and writing (review and editing). EN: analysis, methodology, and validation. X-KY: analysis, methodology, and validation. PA: writing (review and editing). SA: investigation and writing (review and editing). DL: project administration. JM: analysis, methodology, and validation. ME: investigation, methodology, supervision, validation, and writing (reviewing and editing). RP: supervision, validation, conceptualization, investigation, methodology, and writing.
